# CrossTalk opposing view: dynamic cerebral autoregulation should be quantified using induced (rather than spontaneous) blood pressure fluctuations

**DOI:** 10.1113/JP273900

**Published:** 2017-12-05

**Authors:** David Simpson, Jurgen Claassen

**Affiliations:** ^1^ Institute of Sound and Vibration Research University of Southampton Southampton UK; ^2^ Department of Geriatric Medicine (925) Radboud University Medical Center, Donders Institute for Brain Cognition and Behaviour PO Box 9101 6500 HB Nijmegen the Netherlands

**Keywords:** cerebral blood flow, cerebral circulation, blood pressure

Suppose you want to take a car for a test drive. You prefer a smooth ride, so you are probably particularly interested in the car's suspension system. Where do you take your car? Will you take it for a ride on a well‐maintained highway, or will you select worn‐down roads with cobblestones, potholes and speed bumps? The answer is clear: you can't test the car's suspension system if you don't challenge it. Now imagine cerebral autoregulation (CA) as our brain's suspension system, dampening out fluctuations in blood flow as blood pressure varies. In everyday life, ‘bumps’ in blood pressure can be caused by standing up after lying or sitting (orthostatic blood pressure changes), by exercise, emotional stress, medication or infection. The magnitude of these transient changes in blood pressure, for example during orthostatic changes, can easily reach 20% of baseline blood pressure levels (van Beek *et al*. 2008).

Dynamic cerebral autoregulation (dCA) is the concept (or construct) that refers to how the cerebral vasculature counteracts these transient changes in blood pressure. It is an essential concept in human physiology because the unique upright human posture also makes our species uniquely vulnerable to orthostatic hypotension. Being upright requires constant physiological adaptation to counteract the effects of gravity on our circulation, which favours the pooling of blood in the lower half of the body – precisely where our brain is not. Multiple factors can influence the body's ability to maintain blood pressure when upright, such as fluid homeostasis, autonomic function, baroreflex function, drugs that affect the cardiovascular system, infection, lower body muscle contraction, exercise or stress. Together these factors create daily challenges for blood pressure stability, or the ‘speed bumps’ in blood pressure that in turn challenge cerebral autoregulation. The maximum decrease in systolic blood pressure upon standing increases from roughly 30 mmHg between 50 and 60 years of age to 40 mmHg in those over 80, but 95% confidence intervals range from 10 to over 70 mmHg in the normal population (Finucane *et al*. 2014). Sudden changes in blood pressure of this magnitude strongly affect cerebral blood flow and cause perturbations in flow that are almost equal in magnitude (Claassen *et al*. 2009). These large challenges might be compared to the spontaneous random fluctuations in blood pressure in adults at rest, which may typically show a standard deviation of about 6 mmHg (Simpson D., unpublished results from 18 min of recording in 20 healthy young adults).

Therefore, if we want to study cerebral autoregulation in an ecologically meaningful manner, i.e. representative of its main purpose in daily life, do we choose to study cerebral autoregulation while it is operating idly in standby mode, while supine or sitting at rest, or do we want to engage cerebral autoregulation by challenging it in a manner that is more representative of when its brain‐protective function is actually needed?

Below, we will present a brief overview of the various methods that have been investigated to quantify dynamic cerebral autoregulation and discuss the complexity of assessing its function, with particular reference to why increased blood pressure challenges might be preferred.

Currently there is no gold standard to assess dCA, neither for the experimental protocol nor for how to process the recorded signals of arterial blood pressure (ABP) and cerebral blood flow (CBF) (Claassen *et al*. 2016). Indeed, the notion that there would be a gold standard to assess CA may be unrealistic given the complexity of the mechanisms and their possible dysfunctions that underlie the dynamic pressure–flow relationship of the cerebral circulation. Presently, two categories of methods can be identified: measurements with only spontaneous fluctuations in ABP that are taken at rest (supine, reclining or sitting), and methods that have been proposed to cause larger changes in ABP. These include the inflation and release of thigh cuffs, lower‐body negative pressure, a cold pressor test, hand‐grip exercise, Valsalva manoeuvre, and sit‐to‐stand and squat‐to‐stand manoeuvres (e.g. Panerai, 1998; van Beek *et al*. 2008; Payne, 2016). Related are tests of neurovascular coupling (mental tests; passive arm movement) and cerebrovascular reactivity (hypo‐ and hypercapnia; acetazolamide) and tests of static cerebral autoregulation (sCA) involving the use of drugs to raise or lower mean arterial blood pressure over extended periods rather than as relatively brief transients.

The advantage that these methods using induced oscillations in ABP may have are twofold. First, as indicated in the introduction, their larger perturbations in ABP and CBF are representative of physiologically and clinically relevant everyday challenges to CA, where CA responses probably have a necessary protective function. Second, these large perturbations allow us to study the CBF response to ABP with increased certainty that there is a causal relationship between ABP and CBF, which is a prerequisite to assessing CA. With smaller challenges, the response may be masked by the spontaneous variations and other sources of noise in the data.

But can we be certain that the CA we assess using large, induced perturbations is comparable to the CA we assess with smaller, spontaneous perturbations?

There is some evidence that autoregulatory responses to relatively large changes in ABP are similar to those resulting from small changes. In a direct comparison between transfer function analysis (TFA) of spontaneous *versus* induced oscillations (squat–stand manoeuvres), the TFA parameters’ gain and phase were similar, with an expected higher coherence for the squat–stand manoeuvres (Claassen *et al*. 2009). Panerai *et al*. (2001) found great similarity in the ratio of increase in CBF velocity over the increase in ABP for a number of different protocols, including spontaneous responses and induced larger ABP changes, when calculated for the mean of the sample. However, the effect on within‐ and between‐subject dispersion of the different protocols was not tested. Robustness of measures, including a well‐defined and narrow range of values in healthy subjects (which can be clearly distinguished from those found in impairment), and repeatability are additional concerns, as is ‘convergent validity’ (different measures deemed to quantify the same physiological construct provide correlated results) (Tzeng *et al*. 2012).

Finally, we must consider the possibility that the CA responses to induced, large changes in ABP and the CA responses to small spontaneous ABP changes could present two different ‘modalities’ of CA. The responses to increased ABP changes may reflect the more basic and constant underlying mechanisms of CA, while CA responses to spontaneous ABP variations may reflect more time‐varying and context‐dependent modulations in CA.

Could enhanced oscillations solve CA's poor reproducibility and poor correlation between CA metrics? Tzeng *et al*. (2012) found a poor correlation between different dCA measures that were obtained during spontaneous variations in ABP. These results provide a challenge to the common construct of autoregulation, as well as the choice of experimental and signal processing methods. In addition to this low correlation between measures, low repeatability of dCA measures was also observed in a recent multicentre study (CARNet 2, Sanders M & Elting J.W., in preparation), and was also found in previous works (Brodie *et al*. 2009; Gommer *et al*. 2010). Considerable changes over time within the same recording have also been noted in estimates of autoregulation at rest (Panerai *et al*. 2003).

Thus the question arises whether assessment of dCA during larger changes in ABP could solve these problems of correlation and reproducibility.

A decrease in the variability of CA measures has been found to be associated with increased ABP fluctuations, both with spontaneous variability (Liu *et al*. 2005) and in ABP challenges (Birch *et al*. 2002; Claassen *et al*. 2009). This might also be expected from theoretical signal processing considerations, given that the signal‐to‐noise ratio in measurements tends to improve as the excitation becomes larger. It is therefore not surprising that, for example, the detection of impaired autoregulation (during the inhalation of 5% CO_2_ in air) was enhanced when ABP variability was mildly increased using pseudorandom inflations of a thigh cuff (Katsogridakis *et al*. 2012).

Increased changes in ABP, however, do not guarantee good repeatability, as shown by Mahony *et al*. (2000) for thigh cuffs. Strong individual difference not only in the mean of autoregulation, but also in the repeatability across recordings made on the same or on different days has been observed (Mahony *et al*. 2000; Brodie *et al*. 2009).

In summary, increased variations in ABP are not a ‘magic’ solution to robustly assess CA, though there is a consistent decrease in CA variability when CA is challenged by increased ABP variations (Birch *et al*. 2002; Liu *et al*. 2005; Claassen *et al*. 2009; Katsogridakis *et al*. 2012). These findings suggest that enhanced ABP oscillations may lead to better reproducibility in studies that look at repeated measures of CA (e.g. before and after an intervention).

One concern with these protocols, however, is the extent to which these measures might affect CA status itself. There are a number of possible factors. Manoeuvres might change breathing patterns and hence CO_2_ levels, which might be exacerbated by increased CO_2_ production during muscular activity. Hypercapnia is known to be a powerful inhibitor of CA, and indeed is commonly used to provoke temporary impairment of CA in many studies of healthy subjects. Some protocols induce powerful autonomic stimulation (e.g. Valsalva, cold pressor) and there is some debate as to the impact of this on CBF as well as on CA. CA responses change depending on the operating point (mean ABP or resistance–area product) at the start of the manoeuvre (Panerai *et al*. 2001; Cipolla, 2009). Changes in cerebral metabolic demand and thus mean blood flow that may also be associated with the imposed challenges may further confound measurements. Another practical problem is that some protocols increase movement artefacts as there is voluntary (e.g. squat–stand) or some imposed (e.g. lower‐body negative pressure) movement, requiring additional care in data collection but even so, often some data is lost. A major practical concern is whether these protocols would be acceptable in a vulnerable population, such as elderly patients or those in intensive care. Repeated sit‐to‐stand protocols were feasible, however, in geriatric patients, including older patients with Alzheimer dementia (Van Beek *et al*. 2010).

Diversity in CA measures between and within subjects challenges our understanding of autoregulation, as well as questioning the methods used to quantify this construct. In the study of CA from spontaneous changes in ABP, it is a common assumption that the system is linear. The implication of this is that the blood flow response is strictly proportional to the size of the blood pressure challenge, i.e. the response to say a 30% change in blood pressure is six times larger than that to a 5% change in pressure. It thus makes no allowance for a possible threshold effect with more vigorous autoregulation following physiologically more important large swings in ABP, than to weak fluctuations where an autoregulatory response may not be required to protect the brain. Linearity also implies that the response to a positive‐going step in ABP is the exact inverse of the response to a negative‐going step. There is some evidence that this may not be justified – with slightly larger CA responses to increases in ABP than to decreases (Panerai *et al*. 2001; Aaslid *et al*. 2007; Cipolla, 2009). Non‐linear methods that do not make these assumptions have also been used (Chacon *et al*. 2011; Kostoglou *et al*. 2014; Marmarelis *et al*. 2016), and allow for responses that vary according to the size and sign of the ABP challenge, but in general these more sophisticated models with more degrees of freedom have not greatly improved the robustness of CA measures.

Before deciding what is the best protocol for assessing CA, what we mean by ‘best’ must be clarified. A number of possible criteria have been considered, for example good repeatability, ability to predict clinical outcomes, ability to identify changes in CA caused by disease and dysfunction (e.g. stroke, brain trauma) or through hyper‐ or hypocapnia, or a well‐defined range of normality. Until we clearly state the priorities, recommendations as to which method to use to assess CA will remain open for debate. Whether the limited robustness of current measures of CA from spontaneous variations reflects a fundamental problem of CA or our still imperfect choice of CA parameter to estimate from the data is still unclear. Given the current challenges in the field, and in the absence of strong evidence in support of using only spontaneous variations, measuring the response to a larger ABP challenge might be expected to provide greater insight into clinically significant autoregulation and prognostic power than the lesser excitations. While the smooth road makes for a more comfortable ride for all passengers, the bumpy track may give a better understanding of the damping (regulatory) system and lead to more exciting places and greater adventures in science.

## Call for comments

Readers are invited to give their views on this and the accompanying CrossTalk articles in this issue by submitting a brief (250 word) comment. Comments may be submitted up to 6 weeks after publication of the article, at which point the discussion will close and the CrossTalk authors will be invited to submit a ‘LastWord’. Please email your comment, including a title and a declaration of interest, to jphysiol@physoc.org. Comments will be moderated and accepted comments will be published online only as ‘supporting information’ to the original debate articles once discussion has closed.

## Additional information

### Competing interests

None declared.

### Author contributions

Both authors contributed to the conception or design of the work; acquisition or analysis or interpretation of data for the work; drafting the work or revising it critically for important intellectual content. Both authors have approved the final version of the manuscript and agree to be accountable for all aspects of the work. All persons designated as authors qualify for authorship, and all those who qualify for authorship are listed.

### Funding

D.S. is funded by the Engineering and Physical Sciences Research Council (EPSRC) (EP/K036157/1).
